# The pervasive role of social learning in primate lifetime development

**DOI:** 10.1007/s00265-018-2489-3

**Published:** 2018-05-03

**Authors:** Andrew Whiten, Erica van de Waal

**Affiliations:** 1 Centre for Social Learning and Cognitive Evolution, and Scottish Primate Research Group, School of Psychology and Neuroscience, University of St Andrews, St Andrews, KY16 9JP UK; 2 Department of Ecology and Evolution, University of Lausanne, 1015 Lausanne, Switzerland

**Keywords:** Social learning, Traditions, Culture, Ontogeny, Development, Juvenile primates

## Abstract

In recent decades, an accelerating research effort has exploited a substantial diversity of methodologies to garner mounting evidence for social learning and culture in many species of primate. As in humans, the evidence suggests that the juvenile phases of non-human primates’ lives represent a period of particular intensity in adaptive learning from others, yet the relevant research remains scattered in the literature. Accordingly, we here offer what we believe to be the first substantial collation and review of this body of work and its implications for the lifetime behavioral ecology of primates. We divide our analysis into three main phases: a first phase of learning focused on primary attachment figures, typically the mother; a second phase of selective learning from a widening array of group members, including some with expertise that the primary figures may lack; and a third phase following later dispersal, when a migrant individual encounters new ecological and social circumstances about which the existing residents possess expertise that can be learned from. Collating a diversity of discoveries about this lifetime process leads us to conclude that social learning pervades primate ontogenetic development, importantly shaping locally adaptive knowledge and skills that span multiple aspects of the behavioral repertoire.

## Introduction

Social learning and culture (Table 1) have been studied in non-human primates since the middle of the last century. A substantial scientific literature delineating these phenomena has since accumulated, spanning a diversity of vertebrate species including mammals, birds, and fish (Hoppitt and Laland 2013; Whitehead and Rendell 2015; Whiten 2017a) as well as insects and other invertebrates (Grüter and Leadbeater 2015). Primatology has often led the way in these advances, and in the present century has delivered a new range and depth of understanding in this field, supported by a diversity of innovative methodologies (discussed further below). These have often delivered satisfyingly convergent conclusions (Whiten 2012; Watson et al. 2018, for reviews), although there is also ample debate about the exact nature and distribution of the varied forms of social learning across different animal species (Tennie et al. 2009; Whiten et al. 2009; van Leeuwen and Haun 2014; Galef and Whiten 2017; Henrich and Tennie 2017).

**Table 1:** Glossary of key social learning concepts

Conformity: adherence to majority behavior overrides personal adherence to an alternative option (Conformist bias: probability of adopting majority behavior exceeds proportion of community showing it).	
Cultural transmission: diffusion of behavior patterns via social learning from others’ actions or their consequences.	
Culture: (a) broad sense—equivalent to “tradition” below; (b) special sense—a communal complex of multiple traditions (Whiten and van Schaik 2007).	
Emulation: an observer replicates the desirable results of another individual’s actions but using a different means to do so.	
Imitation: an observer copies the form of the actions of another individual.	
Local enhancement: an observer’s attention is drawn to a particular location by the actions of another individual.	
Social learning: learning from others: more specifically, “learning that is influenced by observation of, or interaction with, another animal (typically a conspecific) or its products” (Heyes 1994). Social learning can occur through various specific processes listed in this table, including emulation, imitation, local and stimulus enhancement, and teaching (Whiten et al. 2009).	
Stimulus enhancement: an observer’s attention is drawn to a particular object by the actions of another individual.	
Teaching (defined functionally): behavior performed at a cost to the teacher, which benefits the developmental achievements of a pupil (for extended definition see Caro and Hauser 1992).	
Tradition: a behavior pattern shared by members of a community that relies on socially learned and transmitted information.	
Horizontal transmission: cultural transmission within a generation.	
Vertical transmission: cultural transmission from parent to offspring.	

In the case of cetaceans, Whitehead and Rendell’s (2015, p. 7) comprehensive review concluded that “Culture … is a major part of what the whales are.” In other words, culture is inferred to pervade the lives of the whales that these authors study, shaping so much of their behavioral repertoires that their lives would be drastically different if social learning did not play such an influential role in shaping adaptive behaviors. Whiten (2017b) made a similar case for the cultural lives of the great apes. In the present article, we review the evidence bearing on more specific hypotheses: that social learning progressively pervades the infant and juvenile phases of primates’ lives; and that it recurs to play an important role in later life events too, notably when individuals mature and disperse to new groups.

Our use of the term “pervades” includes a suite of hypothesized effects: (i) that much of the behavioral repertoire is adaptively shaped by learning from others; (ii) that this spans multiple behavioral domains, from foraging to social behavior; and (iii) that effects may span multiple consecutive generations of traditions acquired by juveniles. The main body of this review addresses these issues below. In the remainder of this introductory section, we indicate the principal outlines of what has been learned about primate social learning and culture more generally, within which the particular dimension of ontogenetic development is to be situated.

Our understanding of this field has been enriched and strengthened by the application of a growing variety of methodological approaches to a widening database of primate species. One important “broad-brush” starting point has been to compare geographically separated communities of the same species, identifying behavioral differences that through exclusion of any apparent genetic or immediate environmental explanations are ascribed to cultural transmission. This approach has now identified multiple putative traditions in all the great ape genera (Whiten et al. 1999; van Schaik et al. 2003; Robbins et al. 2016) and in several genera of monkeys (Panger et al. 2002; Leca et al. 2007; Santorelli et al. 2011). More recently, this approach has focused more minutely on differences between neighboring communities of the same species both in enclosures in sanctuaries (van Leeuwen et al. 2012, 2014) and in the wild (Luncz and Boesch 2014), thereby further minimizing the possibility that the behavioral differences identified are caused by genetic or ecological variation.

Such conclusions have been re-inforced by “diffusion experiments” in which alternative techniques to deal with the same foraging task have been seeded in individuals acting as potential models in each of two or more groups, and the subsequent differential spread of these documented, again in both apes (Whiten et al. 2005) and monkeys, both in captivity (Dindo et al. 2009) and in the wild (Gunhold et al. 2014; van de Waal et al. 2015). These experiments confirm a capacity for the transmission and spread of innovations through social learning. Sophisticated statistical approaches delineating social networks have also been used to trace the diffusion of naturally occurring innovations along lines predicted by social relationships (Hobaiter et al. 2014). Transmission across multiple generations has been documented by archeological evidence of nut-cracking excavated deep beneath the surface where the practice continues today, corresponding to over 4300 years for chimpanzees (Mercader et al. 2007; see Fig. 2 in Whiten 2017a) and 700 years for capuchins (Haslam et al. 2016). These studies have been complemented by diffusion experiments running along a chain of individuals where having learned from A, individual B becomes the model for C and so on, thus simulating repeated inter-generational transfer in these genera (Horner et al. 2006; Dindo et al. 2011). Further extensive series of experiments have probed the particular social learning processes or mechanisms employed by monkeys and apes, often focusing on those that appear the most cognitively challenging, notably emulation, imitation, and teaching (Table 1) (Voelkl and Huber 2000, 2007; Subiaul et al. 2004; Call et al. 2005; Dell’mour et al. 2009; Whiten et al. 2009; Hopper 2010; Tennie et al. 2010; van de Waal and Whiten 2012; Galef and Whiten 2017). More recent developments have begun to address selective, adaptive biases in whom to copy, and when (Haun et al. 2012; Price et al. 2017), as well as the constraints imposed by factors such as the relative rank and tolerance of different models and potential learners (Lonsdorf et al. 2016).

In sum, a substantial diversity of methodological approaches has been applied to a growing array of species across the primate order. The now voluminous primate social learning literature, of which the above cited papers offer but an illustrative sample, have demonstrated a significant role for social learning across many behavioral domains, including diet choice, foraging techniques, tool use, predator avoidance, grooming styles, courtship gambits, vocal communication, and reconciliation behavior, plus cross-generation transmission of local traditions.

**Fig. 1:**
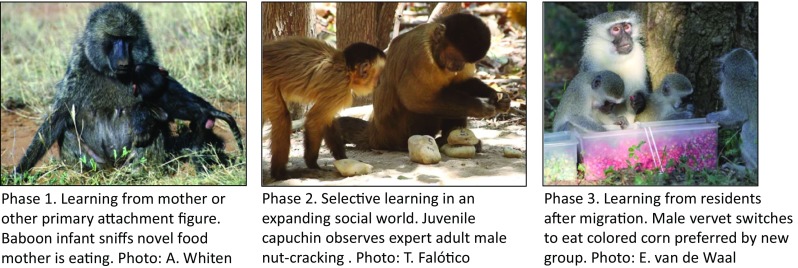
Three proposed major phases in the ontogeny of social learning in monkeys and apes. For full explanation, see text

Within this body of work, attention to developmental dimensions has been just one component. However, we believe sufficient material has now accumulated in diverse pockets of the primate literature to merit and sustain what we believe is the first wide-ranging review of the field, complementing an earlier developmental review focused only on the great apes (Russon 2003). We structure this review in relation to three major phases we suggest can usefully be distinguished in the ontogenetic course of social learning as it unfolds in a majority of primates, illustrated in Fig. 1.

## The first phase of social learning: “mother knows best” (and in some primates, other primary caretakers)

In most species of monkeys and apes, mothers initially carry and breastfeed their infants. This appears a common pattern in primates, although it is not true of all: for example, in callitrichids, infants may be predominantly carried by the father and other family members (a topic treated further below) and some Strepsirrhine primates initially leave the infant in a nest. Nevertheless, in the majority of primates maternal care and carriage is initially the norm, even if the extent to which maternal interactions dominate and thus shape opportunities for social learning varies. One extreme is well illustrated by orangutans, whose typical, semi-solitary social structure means that for as much as the first 10 years or so, the mother is the primary and frequently only model for social learning, sometimes with the accompaniment of an elder sibling (van Noordwijk et al. 2009). Juveniles spend most of their time in the same tree as their mother until they are 6–8 years of age. Even for infant chimpanzees, who will typically experience a greater variety of conspecifics in small fission-fusion parties, a majority of their time will be spent in a focused relationship with their mother. By the age of 4 years, they are still spending most of their time within 3 m of their mother and only around 6% (males) or 3% (females) of their time traveling independently beyond 15 m (Lonsdorf et al. 2014).

Some of the most detailed observational studies of this phase have been achieved in the context of the relatively exclusive mother infant relationship of orangutans, mentioned above. For example, Jaeggi et al. (2008) tested whether the principal function of mother-offspring food-sharing is (a) to provide nutrition or (b) to gain adaptive information about foraging. The authors concluded that their results favored the informational hypothesis, because sharing failed to peak at weaning as the nutritional hypothesis would predict; the article was accordingly entitled “begging for information.” Jaeggi et al. (2010) additionally recorded substantial variance in the diets of multiple mothers, with the dietary profiles of infants found to be essentially identical to their mothers’ profiles; immature individuals focused attention on the most difficult of the mothers’ techniques, and then tended to practice these rather than manipulating other objects, indicating observational learning of the skills involved. Schuppli et al. (2016) labeled such focused visual attention “peering,” in which an infant may bring their face up close to the activity of interest. Building on the studies by Jaeggi et al. (2008, 2010), Schuppli et al. showed that a quantitative index of the complexity of maternal food-processing actions predicted this close peering behavior, especially when the food source was a rare one. Peering was typically followed by a juvenile’s actions on the same target items in the hour following. In parallel fashion, peering at maternal nest building was recorded over the peak acquisition period for nest-building skill, and such peering was followed by a rise in nest-building attempts in the subsequent hour. van Noordwijk et al. (2009) also observed youngsters beginning to perform nest-making actions while their mother made her nest, long before the youngster could make its own nest. As authors of these studies concluded, all the quantified observations logged are those predicted by the hypothesis that observational learning pervades a young orangutan’s construction of its foraging preferences, food processing and other skills, including nest building.

Primate studies that investigate social learning in this way across a breadth of foraging and nesting activities appear to remain rare. However in chimpanzees, in which infants’ early experiences are more maternally focused than one might expect from chimpanzees’ general sociability (Inoue-Nakamura and Matsuzawa 1997), a study of one specific form of tool use has been telling. Young female chimpanzees spend significantly longer periods observing the termite fishing of their mother than do their male siblings, and these females go on to master the requisite techniques as much as a year earlier than their male peers (Lonsdorf et al. 2004; Lonsdorf 2005, 2006). This difference is likely to be of functional significance, since when females reach adulthood, tool-assisted insectivory plays a more important role in their diet than for males, who gain more animal protein from hunting other mammalian prey (McGrew 1979). As in the orangutan analyses, these are correlational findings, so the conclusion that most researchers draw, that they indicate social learning, needs to be tempered by the possibility of a genetic mother-offspring link, such as through biases in manipulative propensities. However, the finding of an even higher mother-daughter matching of the length to which stem tools are inserted into termite mounds (a fidelity tellingly not found for the male offspring that have displayed less peering at the termiting process) are harder to reconcile with an effect of genetic inheritance (Lonsdorf et al. 2004). A now very large corpus of experimental and other studies demonstrate a motivation and a capacity for social learning in young apes consistent with these results from the wild (reviewed in Whiten 2017b, c). Matsuzawa et al. (2008), in a graphic phrase, described such acquisition of skills in chimpanzees as a system of “master and apprentice.” In monkeys living in the wild, selective attention by juveniles has been documented in some detail in white faced capuchins and as in the orangutan studies, found to be focused on relatively rare, large and difficult to process foods (Perry and Ordoñez Jiménez 2006; Perry et al. 2017). Moreover in monkeys, the proposed causal role of social learning has begun to be more directly and rigorously tested by field experiments. van de Waal et al. (2013) studied wild vervet monkeys in several groups that once a month were provisioned with a box of maize corn in order to reveal monkeys’ ranks and alliance dynamics. For the social learning experiments, the corn was divided into two boxes presented side by side and died either pink or blue, with one color of corn having an additive that made it too bitter to eat in two of the four groups studied, and the other color so treated in two other groups. It took three trials at monthly intervals for the monkeys to learn to avoid the locally unpalatable color. This was done around the birth season so that the new crop of suckling infants were not yet feeding on such solid food so did not test it. After a 4-month follow-up period allowing infants to mature, the same pink- and blue-colored corn options were presented again but now with no additive, so it could be tested whether infants who were now starting to eat solid food learned what to eat by trial and error exploration or were instead biased by maternal preferences. The answer resoundingly confirmed the latter, with 26 of 27 infants starting to take the color preferred in their group. The mother of the other infant was of very low rank, so fed on the alternative food box while higher ranked animals continued with their now long-standing preference, and this infant preferentially took corn of the same color as its mother. Accordingly, 27 of the 27 infants ate the option their mothers ate, even though both colors of corn were now equally palatable.

In another experiment, groups of wild vervet monkeys were provisioned with sand-covered grapes (van de Waal et al. 2014). Mothers adopted one of four different techniques to clean them (such as rubbing the grapes in their hands or rubbing them on the ground) and infants showed a significant matching to the technique displayed by their mother. An earlier report showed that such differences are correlated across matrilines (van de Waal et al. 2012), suggesting that the preferred techniques tend to pass down vertically along these kin lines.

A parallel study concerning a very different behavior may reflect very similar social learning in Japanese macaques. Tanaka (1995) conducted fine-grained analyses of video records of the precise way in which mothers removed the eggs of lice from body hair during grooming. Groomers need to free the egg and its ring of cement so that it can be slid up and off a hair and this was done using four different kinds of manipulative configuration, such as using a finger nail to initially scrape the egg loose before sliding it up a hair, or using a “thumb-jig” to free it before removal. Echoing the vervet results, these styles were found to characterize whole matrilines. Again it might be suspected that genetic inheritance could explain these findings, but evidence against this is that from time to time, the preferred technique changed. In one such case studied in detail, a matriarch was observed to change her technique and her daughters and granddaughters soon followed her in this switch, indicating a social learning effect (Tanaka 1998). Tanaka suggests that such changes imply imitative learning of the actions involved. We postpone to below any in-depth consideration of the psychological processes involved in the social learning of these young primates.

Some primates, most significantly callitrichids, deviate from the above picture of an initially primarily maternal context for social learning. In common marmosets, for example, the father typically begins to carry the normally twin infants from birth and later starts to share food with them, while the mother is more limited to the necessarily high burden of lactation for these twins. Other individuals in the group, who are reproductively inhibited, may also care for the infants in what is described as a cooperative breeding system (Schiel and Souto 2016). The corresponding context for early social learning has been examined through complementary observational studies in the wild and experimental investigations in captivity. In the wild, Schiel and Huber (2006) found that observation of adult or subadult foraging (which principally involves locating, catching and consuming invertebrate prey) peaked in 3–4-month-old infants, occurring in close to 50% of all 10-min observation bouts. Half these cases resulted in “model-dependent foraging” in which infants responded within 10 s of watching a model. This took one of three main forms, involving either manipulating the same object within 5 s, foraging closer together, or approaching and acting synchronously, as in feeding on the same food source. Older juveniles of 5–10 months observed others at lower frequencies as they became generally more competent hunters, but when they did attend to others’ actions, they were still likely to then perform model-dependent foraging.

The role of social learning in marmosets was more systematically investigated through experiments in captivity, which contrasted conditions allowing or preventing observation of a model, in naturalistic foraging challenges that involved catching relatively large prey (grasshoppers or crickets) or extracting embedded prey from within covered holes (Dell’mour et al. 2009). Infant observation of adults (their mother, in this study) peaked at a similar age to that earlier recorded by Schiel and Huber (2006) in the wild, and these infants were 15 times more likely than non-observers to tackle the problem presented. They also needed significantly fewer trials to achieve mastery. Infants were able to successfully catch and kill insect prey within 5 months so long as they observed their mother hunting the same species. Further below, we discuss whether modifications of parental behavior in this context may represent a simple form of teaching.

The pattern of early social learning focused on primary caretakers is also apparent in the human primate, from feeding behavior to language acquisition. For example, Hewlett and Cavalli-Sforza (1986) conducted in-depth interviews with Aka hunter gatherers in the Central African Republic, asking from whom each of 50 very different skills, ranging from foraging to food sharing to infant care, had been learned. Respondents were reported to offer detailed descriptions of whom they watched performing the skill or the few things the person said to transmit the skill knowledge. From these responses, the authors concluded that “unquestionably, parents are the primary contributors” (Hewlett and Cavalli-Sforza 1986, p. 928), their average contribution being reported as 81% overall, and as much as 89% on average in the case of food-processing skills. These figures might be somewhat inflated in favor of vertical transmission through the self-report methodology (Aunger 2000), but Aunger’s own data based on inter-household versus inter-clan cultural similarities in food taboos concurred in describing an initial phase of cultural learning from parents.

These results echo a general conclusion with which we close this section, namely that for juvenile non-human primates, it is crucial to have mastered subsistence skills sufficiently well to sustain the independence required by the age of weaning, and the primary caretaker or caretakers, typically the mother, are those providing the main models. In the wild the importance of the latter derives from the fact that what may be a complex dietary array needs to be selected from a massive range of potential options in the natural environment that vary much in their nutritional payoffs as well as being noxious or toxic in many cases. Over a year, a community of chimpanzees may exploit over 300 different kinds of food items, including only certain parts of plants such as the peeled pith, the peel itself being toxic; in Lope, Gabon, for example, fruit alone is harvested from 114 different plant species (Inskipp 2005). The preferred items are selected from among hundreds if not thousands of alternative species and parts (flowers, fruits, pith, storage organs). A similar task is faced by gorilla and orangutan infants (Whiten 2017b) and to a greater or lesser extent, all primates.

Given such complexities and dangers in primate feeding niches, trial-and-error learning is likely to be inefficient, if not overtly dangerous given the distribution of poisonous elements adapted to deter consumption, whereas social learning instead taps an existing knowledge base of the community. The importance of social learning may nevertheless vary according to food type. In a preliminary study of howler monkeys, Whitehead (1986) noted that in the case of mature leaves, that often contain toxins, mothers would often wait until their infant joined them before selecting leaves to eat, and infants always waited for adults to feed first and observed them. By contrast in the case of fruits, that depend on being eaten for seed dispersal, and so are more rarely toxic, infants were more likely to initiate their own feeding activities (Whitehead 1986).

The most relevant of the knowledge transmitted may be significantly localized, making learning from a mother familiar with the locality important: for example, in comparison with intra-population homogeneity, 60% of the dietary preferences of orangutan populations on either side of a large river were found to be different (Bastian et al. 2010). In a recent review, Whiten (2017b, p. 7793) suggested that “years of close apprenticeship to a mother who daily displays her knowledge of such a large but selective diet-set likely provide an important means of achieving an adaptive response to this challenging complexity.” Schuppli and van Schaik (2017) used the metaphor of an iceberg to describe this situation: they suggest we have tended initially to identify only the iceberg’s most visible “tip” of socially learned repertoires, especially salient items like tool use, neglecting the greater proportion of more mundane behavior such as what to eat, where to sleep, and what are things and places to beware of. Relatively, simple social learning processes, such as stimulus and local enhancement of the relevant items, or overt negative responses to them, may suffice to permit much of this scale of information acquisition, whether in visual, vocal, or olfactory modes (see Fig. 1).

## Widening circles of influence: “learning from the best nutcrackers” and other functional biases

In the case of human childhood, Henrich and Broesch (2011, p. 1140) propose “a two-stage learning model in which individuals first acquire information from their parents … and then later update this information based on information from their preferred models.” These authors provide a range of lines of evidence supporting this basic model from a field study in small-scale Fijian villages, where in the second stage proposed above, individuals begin to obtain information from those judged better models than their parents for specialist activities such as fishing, growing yams or medicine. This basic two-phase model maps to what we proposed earlier in this paper for non-human primates: initial learning from primary caregivers, typically the mother (as reviewed in the section above), followed by a progressively widening circle of learning from others (Fig. 1). Experimental evidence consistent with a developmental shift from an initial preference of children to learn from parents to models with alternative expertise has come from controlled studies of both acquisition of manipulative expertise (Lucas et al. 2017) and trust in verbal informants (Harris and Corriveau 2011). In non-human primates, the quantitative study of young orangutans’ peering behavior mentioned earlier (Schuppli et al. 2016) showed that by about age 5, close to weaning, peering at the mother tipped below 50% and became focused more toward others from whom there may yet be something new to learn.

Henrich and Broesch (2011) predict the second of the two broad phases to be selective, and they propose and provide evidence from their Fiji studies for a suite of such learning biases, all of which are argued to achieve adaptive outcomes. Emphasizing such inferred functionality, these biases have been referred to in comparative research as “social learning strategies” (Laland 2004), although labeled elsewhere by other, more neutral terms like “transmission biases” (Boyd and Richerson 1985). Recent years have seen an escalation of published reports about these biases in humans, non-human primates and other species (Rendell et al. 2011; Hoppitt and Laland 2013; Price et al. 2017). As yet, only a small proportion of this work has a developmental focus in non-human primates, but sufficient studies are now available for us to address a number of the biases listed by Henrich and Broesch (2011), and we shall add further to these. In what follows, for brevity, we describe selectivity in terms of biased “copying” but we do not necessarily imply high-level processes like imitation by this: if a juvenile is biased to eat what dominant individuals eat, for example, we might express the rule as “copy high rankers” even though the process may be as simple as stimulus enhancement of a particular food type.

Henrich and Broesch’s (2011) first and arguably most important bias is “perceived success or knowledge.” As an example, they found that in Fiji, believing someone to be among the best spear-fishers increases by a factor of 10 the chances that such experts will be chosen to learn from 2 years later. For medicinal plant knowledge, the bias rises to a factor of 25. Their finding that perceived success was more influential than inferred knowledge is worth highlighting because non-human primates can in principle judge a potential model’s success by direct behavioral observation. A clear primate example is indicated by one paper’s title, “Watching the best nutcrackers: what capuchin monkeys know about others’ tool-using skills” (Ottoni et al. 2005; see also Coelho et al. 2015; and see Fig. 1). These authors reported that close observation of stone-tool-based nut cracking is prevalent in young capuchins and that the latter preferentially target the most proficient (and not just the most active) nutcrackers. Nut-cracking adults are tolerant of this close attention and indeed permit scrounging, which occurs in 35% of cases, so this could be the immediate causal explanation for the phenomenon. However, the authors highlight that “This simple mechanism could, by itself, optimize the conditions for the social learning of nut-cracking techniques and for the diffusion of tool-aided nut-cracking as a behavioral tradition” (see also Fragaszy et al. 2017). Indeed in marmosets, Caldwell and Whiten (2003) showed through controlled experiments that such scrounging may facilitate social learning of foraging behaviors. Other experimental studies have demonstrated that chimpanzees will discriminate and copy the choices of group-mates who are foraging faster at a resource-rich site than those at a site delivering a lower rate of payoffs (Vale et al. 2011; see also Brosnan and de Waal 2004, for capuchins), and Barrett et al. (2017) provided evidence of preferential copying of proficient extractive foraging individuals in white-faced capuchins. However, none of these three studies specifically targeted juvenile subjects. In an artificial foraging task, Kendal et al. (2015) found that chimpanzees were biased to copy models described as “knowledgeable” rather than simply discriminating success: these preferred models were those who had been trained to succeed, and the authors speculated that what observing chimpanzees may thus have discriminated was these individuals’ confident and purposive approach to the task, given that other potential models were just as successful. However, this study also did not target juveniles as observing subjects, and we look forward to more studies on this topic that do so.

Henrich and Broesch (2011) also found a bias to learn from older models, model age thus likely acting as an indirect predictor of the best individuals to learn from. Reflecting a similar bias, in field experiments introducing novel nuts to nut-cracking chimpanzees in the wild, Biro et al. (2003, p. 213) found that juveniles were “highly specific in their selection of conspecifics as models for observation, attending to the nut-cracking activities in the same age group or older, but not younger than themselves.” Similarly, Barrett et al. (2017), after introducing a new hard-shelled fruit to wild white-faced capuchins, reported a similar bias to observe models older than oneself.

A bias to prefer one sex of model over the other was also investigated by Henrich and Broesch (2011), given the division of labor common in Fijian societies. It was found that all subjects were biased to prefer males as models in relation to fishing and yam cultivation, whereas female models were preferred for medicinal expertise. One area where one might expect related biases in primates is when young male primates may need to learn male-related skills that they cannot learn from their mother. In one such example, wild male tufted capuchin monkeys were found to eat more animal foods and forage more for invertebrates along large branches, while females ate more fruits and fed more on leaves and bamboo microhabitats (Agostini and Visalberghi 2005). Correspondingly, juvenile males were found to progressively spend more time with male adults, focusing their food-related attention on them and eventually adopting the typical male array of foraging preferences. In similar fashion but in a different study, only male capuchins acquired stick-probe use, with young males preferentially observing older male experts (Falótico and Ottoni 2014). Mörchen et al. (2017) confirmed the earlier observation of Schuppli et al. (2016) that young orangutans showed a clear dependence on peering at their mother’s activities, whereas as they developed, older individuals showed a preference for watching immigrant unflanged (not fully mature) adult males’ activities, especially in the nesting and social context. The authors speculate that unflanged males may thus act as cultural vectors, facilitating the transfer of traditions between orangutan populations.

In some cases, the functional reasons for an attentional bias to one sex may not arise from diet divergence so much as local expertise. In experimental tests of learning to open an artificial fruit by wild vervet monkeys, van de Waal et al. (2010) found there was evidence for social learning only when the model was an adult female. This may make functional sense insofar as females are permanently resident in their ranges while males disperse, so females are likely to be the local ecological experts to preferentially learn from. However, this study did not focus on juvenile observers of these female models.

A final bias not considered by Henrich and Broesch (2011) (perhaps surprisingly given the many theoretical and modeling studies of Henrich on this topic) is conformity—copying a majority of one’s group. Perry (2009) painstakingly logged the frequencies of young white-faced capuchins watching either of two different ways of processing Luehea fruits (pounding versus scrubbing) over their first 5 years, starting with mothers and extending to others and found that individuals tended to adopt whichever technique they had witnessed occurring with the greatest frequency overall.

All of the above biases are conceptualized as preferences of the learner. However, whom a growing individual may learn from will also be constrained by the tolerance for close proximity by the potential model. This varies between species (van Schaik et al. 1999; van Schaik 2003), and also in relation to intra-specific learner-model pairings, graphically illustrated by Russon’s (2003) tabulation of over 50 such potential permutations of age-sex classes in orangutans. Both inter-specific and intra-specific variations may shape constraints on, and opportunities for, social learning.

## A life-long ontogenetic perspective: social learning at the time of dispersal

In the above, we proposed two initial phases in the ontogeny of primate social learning: a first focused on the primary caretaker, in most species the mother, and a second characterized by progressive learning from a widening social circle in an individual’s group. Here, we address a third phase that may occur on dispersal from one’s natal group, typically an activity that involves males in some species and females in others, avoiding inbreeding. On dispersal, an individual will experience a new physical territory and a new social context. Each of these will likely bear some resemblance to the natal array, but may differ in others, and will certainly do so in important details, all of which potentially creates a significant further phase in which social learning from residents may be beneficial. For example, the migrant individual will initially know nothing about where important foraging, drinking and sleeping sites are, and the foraging spectrum may even include new food types and associated foraging techniques (Russon 2003). On the social side, there may be much to be learned about local social dynamics, as for example, whom to respect for their high rank. Alternatively, a migrating individual may be the possessor of skills not yet present in their new group, so in this case, it is residents who may learn from the immigrant, who acts as a tradition bearer from its natal culture.

In the course of the experiment described earlier that used trained group preferences for eating pink or blue corn to test for social learning in infancy, as many as ten male vervet monkeys happened to conduct their dispersal so they moved from a group that mostly ate one color of corn to one that habitually preferred the other color (van de Waal et al. 2013). With a surprising degree of alacrity, all but one of the ten adopted the local preference as soon as they were not outranked at the food source and were free to decide which color to eat (see Fig. 1), a switch also found in avian cultural diffusion experiments where birds similarly dispersed between ranges in which different foraging behaviors had been experimentally created (Aplin et al. 2015). A similar switch to behavior matching that of residents has been described in chimpanzees living in neighboring ranges of the Tai Forest where details of their nut-cracking techniques differ (Luncz et al. 2012). Females transfer between these communities, yet come to behave as do the residents, which in one community involves a year round preference for stone hammers that occurs only seasonally in two others (Luncz and Boesch 2014; Luncz et al. 2015). Similarly, a female chimpanzee migrating to a neighboring community displaying a different style of hand-clasp grooming tended to conform to the new local habit (Nakamura and Uehara 2004).

All these cases appear to reflect a disposition to abandon existing personal preferences or behaviors and instead conform to the new local norms. One possible functional explanation is that such a disposition is adaptive in a context of uncertainty about what are the optimal local foraging behaviors to utilize, a good guide to which is offered by the existing residents. A second and quite different adaptive explanation is that by matching the behavior of residents, an incomer may be better accepted into their new group (and social affiliation with those who copy one’s behavior has been experimentally demonstrated in macaques by Paukner et al. 2009). At present, it seems not possible to clearly distinguish between these two explanations, but in the case of the vervet monkeys, further ongoing tests in overlap ranges that males would already be familiar with may show whether the first, ecological explanation can be discounted if conformity occurs in such regions.

Conformity in social behavior is less likely to be consistent with an ecological explanation in any case. Evidence for one such adjustment came in a study of wild baboons in which stealing of infected human food by the most dominant males led to their death (from TB), engendering low levels of aggression in the group. Sapolsky and Share (2004) presented evidence that in later years, as new males entered the group, the peaceful tenor was maintained and hence described as a “pacific culture” adopted by the immigrants.

Cases of the alternative scenario in which instead, immigrant behavior prevails and is adopted by residents appear rare. A case where the inference that this must have happened in the past is offered by nut cracking in chimpanzees. This occurs only in an area spanning about 500 km in West Africa and not elsewhere in the entire range. It has been identified in at least eight communities across that Western region (Carvalho and McGrew 2010). Presumably, it must have spread through the dispersal of mainly female culture bearers. When Biro et al. (2003) introduced a new nut species into one of these communities, the nuts were cracked only by a chimpanzee who had migrated from a region where these nuts were already known and cracked. Her practice was progressively adopted by other members of her adopted community, although this process took several years to play out. In one case the technique of ant-fishing spread in a chimpanzee community in which it had not been seen over decades of prior study, following the immigration of a female from a community in which the behavior was habitual (O’Malley et al. 2012).

## Socio-cognitive transmission processes in primate ontogeny

The principal focus of the present review is on the role and scope of social learning in the behavioral ecology of developing primates, irrespective of the underlying mechanisms. However, just what a juvenile primate can acquire by observation, given the particular social learning capacities at its disposal, will constrain its adaptive flexibility. A primate that can copy adults’ foraging or tool-use skills through a process such as imitation is in a different adaptive situation compared with one that cannot, and is instead restricted only to such simpler processes as stimulus enhancement, that focus its attention on relevant entities such as the optimal objects to feed on. Accordingly, we here offer an overview of some core relevant findings. Table 1 lists some of the principal psychological processes underlying primate social learning, investigations of which have been reviewed in recent years by Whiten (2012), Whiten (2017a, b, c) and Galef and Whiten (2017).

As those reviews confirm, research on primate social learning, which now spans over a century of work, has generated a voluminous literature. This includes a large proportion of laboratory-based studies because these are best able to implement the necessary control and individual testing conditions. For varied and often practical reasons such as subject availability, infants and juveniles figure relatively infrequently as subjects, despite the evidence reviewed above that it is in juvenile phases of the life history where social learning is likely to be particularly prevalent. There are also marked species biases, with a large preponderance of research on chimpanzees, often making comparisons with social learning in our own hyper-cultural species (Galef and Whiten 2017; Whiten 2017c). These biases come together in the fact that a suite of influential experimental studies has documented cultural transmission of alternative tool-use and other techniques spreading within and even between chimpanzee communities, but these have been largely composed of adults (reviewed in Whiten 2012).

The now extensive corpus of experimental studies dissecting social learning processes have principally focused on whether imitation, defined as copying the form of another’s actions (Whiten and Ham 1992) and assumed to be the most complex and/or specialized process, is in operation, or some simpler alternative. The latter include stimulus enhancement and local enhancement, which draw the attention of the learner to particular objects or locations respectively, and emulation, in which an observer learns about the environmental results of actions rather than the form of the actions themselves (Table 1). Perhaps bizarrely, relatively little experimental work has accordingly focused specifically on the supposed simpler processes, despite the real possibility that they may play the major role in much of juvenile primates’ social learning in the wild. The findings we reviewed indicating extensive social learning about what species, and which parts of them, to eat, require only a role for stimulus enhancement, while local enhancement could engineer learning about beneficial foraging locations, sleeping sites and associated travel routes. Much circumstantial, correlational evidence is consistent with this as reviewed in earlier sections of this paper, yet the only field experiment directly testing such effects we are aware of is our own, in which as described earlier, mothers were trained to prefer either pink or blue corn, a preference their infants did indeed follow faithfully when they began to sample these foods (van de Waal et al. 2013). Scrounging food scraps from the mother or others may help funnel infants’ focus on the selectivity of experienced models, as shown by experimental tests (Caldwell and Whiten 2003). However, given that several studies with captive primates have reported a lack of such discrimination (Fragaszy et al. 1997), more tests in the wild are needed to clarify whether such apparently conflicting findings reflect the effects of captive rearing (discussed further by Perry and Ordoñez Jiménez 2006).

The enhancement effects outlined above have a positive valence (i.e. are positively valued by the animal concerned), which may also apply to domains other than foraging, drinking and sleeping, such as in mate choice copying, for which there is evidence in fish (Dugatkin 1996). However, the corresponding experiments needed to test such effects are rather intractable in primates. Other enhancement effects may have negative valence (i.e., be actively avoided by the animal). The most obvious functional example and perhaps the most critical one is avoidance of predators, where laboratory experiments have shown juvenile macaques quickly developing fear responses to objects that their mother showed fear of (Mineka and Cook 1988; see Russell et al. 1997, for chimpanzees). In an apparent parallel in the vocal domain, Cheney and Seyfarth (1990) described how juvenile vervet monkeys, although apparently having innate biases to use different alarm calls for aerial and terrestrial predators, nevertheless showed a progressive convergence on the specific targets eliciting alarm calls by experienced group members, initially calling when sighting (harmless) vultures but later ignoring them, whereas the response to martial eagles, with which adult calls are associated because they are the true danger, became the strongest, suggesting learning from these experienced adults. Equivalents to such negative valence in non-predator contexts such as foraging appear less prevalent. One potential example comes from observations on a mother chimpanzee responding to her infant reaching for leaves of a non-food tree: “her mother, FT, took PN’s hand and moved it away from the leaves. As PN continued … FT took the leaves from PN’s hand, plucked all the leaves within her arm’s reach and dropped them to the ground” (Haraiwa-Hasegawa 1990, p. 280). Other mothers behaved similarly and they “prohibited … infants only from feeding on the individual trees that they themselves never fed on”.

Turning to focus on the role of imitation in primate development, it is generally assumed that this is the most cognitively complex of the social learning processes. This is because imitation requires the transformation of forms of action by others that are perceived in some sensory modality (the visual modality being most analyzed, but imitation can also refer to vocal copying) into appropriately matching motor outputs by oneself (Whiten and Ham 1992). Imitation is also often assumed to permit the highest fidelity of transmission of action patterns, thus providing strong support to the spread and maintenance of cultural traditions, and in the view of numerous authors, key in the emergence of human cumulative culture (Tomasello et al. 1993; Henrich and Tennie 2017). Both cumulative culture and imitation itself have been argued to be limited only to our own species (Tennie et al. 2009). Such conclusions assert that non-human primates’ most complex social learning is limited to emulation, characterized by learning only about the environmental results of actions rather than the actions themselves.

However the imitation-emulation dichotomy is not so clear as at first sight. It is not straightforward where the boundaries of “actions” that may be copied (“imitation”) lie. One criterion some authors adopt is that only *bodily* copying counts as imitation (e.g., Tennie et al. 2012). But when a tool is used, it becomes effectively an extension to the body, so is copying the form of a tool’s movement, as in, for example, poking versus levering, emulation, or imitation? Perhaps copying such movements may have similar cognitive requirements to copying the form of particular body movements and have similar implications for the faithful transmission of cultural patterns. In such contexts, both imitation and emulation may be involved—and beneficial in copying the “gestalt” of the bodily and tool movements and their effects. Similar considerations can be extended to the form of the changes a tool or a hand may effect on an object such as a fruit, so we may envisage a continuum in the causal sequence of bodily and environmental happenings that may be copied, possibly with associated tool-based happenings in between.

Just what parts of this causal cascade of bodily and external happenings are copied (and in particular whether details of bodily movements are imitated) may not necessarily constrain the long-term life of a tradition. For example, we have archeological evidence that tool-based nut-cracking by chimpanzees has been transmitted for over 4000 years (Mercader et al. 2007), a long period of faithful transmission compared with most contemporary human traditions one can think of, and we also have experimental evidence that the transmission of this skill to juveniles rests on social learning (Marshall-Pescini and Whiten 2008; Whiten 2015). High fidelity motor matching may not be essential to such cases (see also Fragaszy and Visalberghi 2001): so long as a rough copy of the hammering action is refined through extensive cycles of practice and observation, and delivers important nutritional payoffs, nut-cracking may well be sustained with adequate fidelity down the ages, as every generation of juveniles copies what they see existing experts do, and confirm it delivers great rewards.

Nevertheless, ghost experiments in which environmental effects are made to occur with no agent visible indicate that seeing another individual *do actions* facilitates learning of their consequences in the more elaborate cases (Hopper et al. 2007, 2008, 2015). Direct evidence that apes can imitate bodily actions, even if with lower fidelity than children, comes from “Do-as-I-do” experiments in which the subject is taught to try to replicate a training set of bodily actions when requested, then tested on a novel battery of manual, facial and gross bodily movements. These were first reported for a young home-reared chimpanzee by Hayes and Hayes (1952), then later replicated with non-enculturated “lab” chimpanzees by Custance et al. (1995) and Pope et al. (2018) as well as with an enculturated adult orangutan by Call (2001). Evidence that chimpanzees observing others are cognitively encoding what they see in terms of actions comes from a case where in one juvenile this “spilled over” the normal inhibition that occurs while watching an act that may later be imitated. This youngster instead acted out the nut cracking actions while watching the older chimpanzee perform, sometimes even in approximate synchrony (Marshall-Pescini and Whiten 2008; Fuhrmann et al. 2014). The youngster had no hammer or nut, so this could not be emulation.

Similarly, young enculturated chimpanzees and orangutans observed a model and, after a 10-min delay, they often replicated the actions the model had performed (Bering et al. 2000; Bjorklund et al. 2000). These studies tested copying of a large range of actions on many different objects, demonstrating matching in such witnessed acts as holding a drill in one hand and turning the crank to make it drill or putting a nail in a form board and using a hammer to hammer it.

Such copying can be selective in ways that may be regarded as rational. Horner and Whiten (2005) showed that young chimpanzees tended to copy all parts of an action sequence used to extract food from an opaque artificial fruit, but when some parts of the action sequence could be seen to be ineffective in a transparent version, these were likely to be omitted from the apes’ own efforts. Children, by contrast, tended to copy these, a response later labeled “over-imitation” (Lyons et al. 2007), which has spawned a now-substantial research literature in developmental psychology, as well as two replications of the ape results (Nielsen and Susianto 2011; Clay and Tennie 2018).

Evidence of bodily imitation in primates is not restricted to apes, although to our knowledge, experimental tests with monkeys have not included juvenile subjects, as the ape research has. Voelkl and Huber (2000) showed that compared with a sample of marmosets that typically used their hands to open an artificial food canister, those who witnessed a model use her mouth were more likely to apply that method. Since this had the same effects on the canister, the difference could not be explained by emulation but rather bodily imitation, even if at a crude level of manual versus oral manipulation. Similar evidence for imitation in birds using either their beaks or feet to produce the same environmental effects have been demonstrated in more than one species of bird (Zentall 2004) and the same mouth/hand copying was replicated for wild-born vervet monkeys in a sanctuary in South Africa by van de Waal and Whiten (2012), followed by spread of the preference for different techniques within models’ respective groups.

In a very different experimental approach, de Waal and Johanowicz (1993) managed to cross-foster two species of macaque, only one of which naturally displays a strong disposition for reconciliatory responses after aggression, and found that such behavior became more common in the monkeys reared with the conciliatory species. It is difficult to see how this could come about by a process that fits the conception of emulation, suggesting it rested on copying the behavioral dispositions of the adult society the youngsters were cross-fostered in.

The transmission of a variety of other behavior patterns in monkeys appears difficult to explain other than by imitative copying. A striking example is what Perry et al. (2003) described as social conventions, in which bizarre habits of pushing fingers into the mouth, nostrils, and even eye sockets of close companions arose, diffused in certain groups, and later faded, in white-faced capuchins, which seem difficult to acquire other than by imitatively mirroring what another monkey does to oneself. In a very different example, Leca et al. (2007) identified as many as 39 different forms of the strange “stone-handling” behaviors of Japanese macaques, different arrays of which were exhibited in different groups, again suggesting copying of the local behavior patterns.

Perhaps surprisingly, emulation behavior has been little tested explicitly, instead tending to be the default explanation offered for social transmission where there is little or no evidence of imitative matching. An interesting exception is an experiment by Tennie et al. (2010), who showed (adult) chimpanzees how to pour water from a bottle into a tube, so that a peanut inside rose high enough to be extracted. Nearly a third of the subjects, who were then presented with a dry tube and peanut but no bottle, took water into their mouths from their dispenser and spat it into the tube to create the same effect, demonstrating emulation in the original sense suggested by Wood (1989) and Tomasello (1990).

Teaching, defined functionally rather than intentionally as any behavior performed at a cost to the teacher that benefits the development of competence in the pupil, has been increasingly documented in a variety of species in recent years (Hoppitt et al. 2008; Thornton and Raihani 2008). In mammals, this is often in predatory species where the young need to make a big leap from nutrition based on maternal suckling to catching and dispatching elusive prey. We suggest that the best evidence for teaching in primates fits this context, as illustrated in the behavior of callitrichid adults that on finding their typical invertebrate prey such as insects, emit vocalizations that attract the young but themselves desist from prey capture, so “scaffolding” the youngster’s initial attempts at predation (Rapaport and Ruiz-Miranda 2002; Rapaport and Brown 2008; Dell’mour et al. 2009). Perhaps the closest to this in other primates is displayed in the tolerance of mothers to allow young to take their tools and food targets, such as in chimpanzee nut cracking (Boesch 2012) and termite fishing (Musgrave et al. 2016). We judge that the suggestion of Hoppitt et al. (2008) that teaching is not as elaborate in chimpanzees as one might expect from the sophistication of some aspects of their social cognition appeals to the functional context: unlike for predatory species, the transition from suckling to foraging on items like fruits can be an easier and more direct one, that can be adequately achieved by observational learning alone.

## The roles of juveniles in primate culture: social learning, play, innovation, and practice

**Fig. 2:**
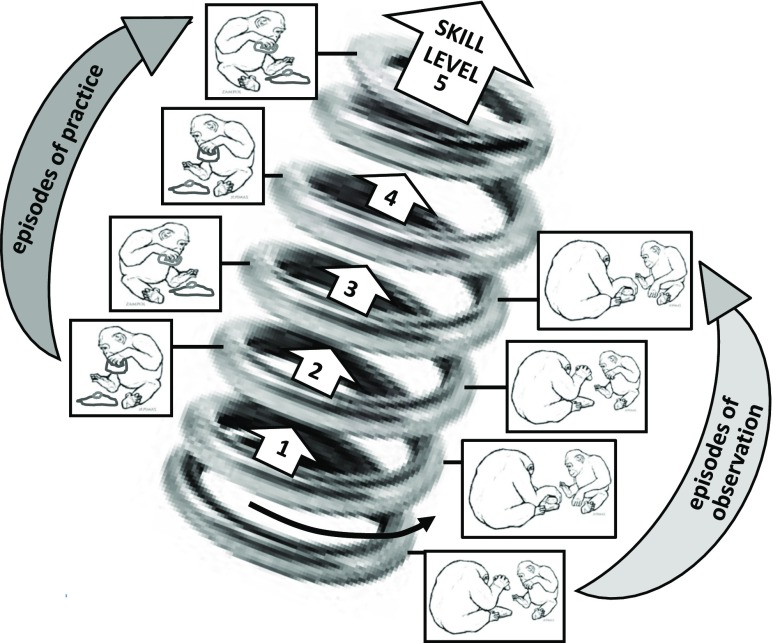
“Helical curriculum” model of social learning of complex skills. Educationalists talk of a “spiral curriculum” in which topics are re-visited at increasingly higher levels—but representing the developmental time dimension creates a 3D helix rather than a 2D spiral. At each turn of the helix, a juvenile watches a model and learns from them. Between such observational episodes is a period of exploration and playful practice, as a result of which the learner is able to extract additional information in consecutive observational periods, including more refined aspects of the skill that the youngster could not assimilate earlier. Corresponding skill levels thence rise progressively, indicated by levels 1–5. Modified after Whiten (2015), Whiten 2017a, b, c)

In a remarkably prescient early paper, “The nature and uses of immaturity,” Bruner (1972, p. 688) suggested that understanding the nature of primate development and in particular the “evolution of educability” requires analysis of both social learning and play, the latter occupying so much of a juvenile primate’s life. Despite decades of research (Bruner et al. 1976; Fagen 1981; Bateson and Martin 2013), the function or functions of play have yet to be compellingly demonstrated, perhaps in large part because play cannot be easily experimentally manipulated, limiting our ability to clearly establish causation with respect to its proposed benefits. Nevertheless, there is something of a consensus among the authors cited above that play provides a form of uniquely flexible (rather than rote) practice. Fagen (1976) proposed an insightful analogy with what engineers can discover by running programs to guide a model aeroplane’s extreme (“playful”) explorations of its actions in a wind tunnel, feedback from which can be utilized to make the program more sophisticated in its response to future challenges that cannot be predicted in all detail in advance. Accordingly, Fagen (1976, p. 99) described play as “optimal generic learning by experimentation.” The essence of Bruner’s linkage of observational learning to this conception of play was elaborated upon by Whiten (2015) in a graphic designed to model the ontogeny of nut-cracking behavior and similar difficult skills in chimpanzees (Fig. 2). Here, following a bout of observational learning, the juvenile applies what it has learned in playful exploration and practice. Then, with the benefits of such actions and the feedback they generate available, the youngster returns to observe an adult model again, now being able to extract more applicable information than before. This cycle continues until the skill is effective and the benefits of further observation attenuate. A further “twist” to this helical model is suggested by Russon’s (2003) point that changes in age-related competencies (and strength) can change an individual’s approach to a task such as complex manipulative or tool-based foraging problems, so these need to be “re-solved” in different ways through development, potentially with the aid of further observational learning from skilled individuals.

Apart from its potential role in individual ontogeny, the innovative aspect of play suggests a potential role for juveniles and their playful explorations in the population-level phenomena of cultural evolution, because innovation is necessary for evolutionary change. Such effects might in principle extend to cumulative culture, in which innovation builds on earlier inventions that have already been incorporated into current traditions. Early and famous examples are due to Imo, the juvenile female Japanese macaque who first invented sweet-potato washing and later wheat grain sluicing, that led to some of the first reports of primate “proto-cultures” (Hirata et al. 2001, for a review). Imo’s inventions first spread to her juvenile peers and eventually to adult females, thence being transmitted vertically down to offspring, in line with the picture we presented in the first section of this review.

Innovation, whether playful or not, has been subjected to little systematic study in the field, perhaps in part due to difficulties in defining and measuring it. However a major and rigorous onslaught on the topic has recently been completed by Perry and her colleagues, in a 10-year study of ten groups of white-faced capuchins, extending to the lives of 234 individuals (Perry et al. 2017). Innovations were defined and recorded in the latter 5 years of the project as those behaviors that no researcher had seen in the group in the prior 5 years, with each of these two periods yielding over 35,000 h of observation. In total, 187 such innovations were identified across the domains of foraging, social, investigative and self-directed behaviors. The majority of these were never taken up by others, with no more than 22% being later socially transmitted. For example, using the tail tip to sponge water out of tree holes arose in four groups over the whole 10-year period but only spread socially in one of them. That a majority of novel behaviors are not necessarily picked up by others echoes the results of a retrospective analysis of records of innovation in Mahale chimpanzees, reporting that only 11 of 32 behaviors never seen in the first 15 years of a 40-year study spread significantly among others (Nishida et al. 2009). These authors conclude that “It appears to be difficult for a new behavioral pattern to propagate from a single newcomer to many members of a society … In contrast, it seems to be easy for a newcomer to acquire an established pattern, as was seen for subadult female immigrants who quickly became habituated to human observers after immigration” (p. 34; see Samuni et al. (2014) for documentation of the latter effect in a different chimpanzee community). Nishida et al. (2009) comments that “from many to single, that is, socialization” may be a relatively easy process, by contrast with the launching and spread of a new innovation. As we remarked in reporting our pink-and-blue corn experiment, this would be the consequence of a social learning bias to copy the majority in one’s community, and perhaps explains why social learning appeared potent in our colored corn experiment, compared with other field experiments that instead seeded new foraging techniques in only single initial models.

Perry et al. (2017) found that juveniles were responsible for a majority of innovations overall, spanning domains of foraging, investigation and self-directed behavior, whereas adults generated more innovations in the social domain, such as the “bond-testing” behaviors involving mutual insertion of fingers into each others’ nostrils and eye-sockets. The authors argue that these biases are functional, as juveniles’ learning and exploration is principally focused on foraging and other survival skills, whereas in adulthood social dynamics, including bond testing through changing social customs, become more critical for reproductive success. The quantitative results of this study thus appear to confirm the early speculations of Bruner (1972) outlined above, that the playful and exploratory mode of primate juvenility plays a significant role in innovation, interacting with processes of social learning both at the individual level (“the helical curriculum”: Fig. 2) and transmission at the broader cultural level (as innovations are necessary to cultural change).

These results do differ, however, from a large-scale survey of the primate literature undertaken earlier by Reader and Laland (2001). Scoring the literature up to this date for records of behavior described as novel or innovative, these authors logged 533 instances, 45% of which concerned foraging. The significant finding for our present discussion is that they reported a majority of innovations by adults rather than juveniles, which as the authors noted, “runs counter to contemporary thinking” (Reader and Laland 2001, p. 801). However, this survey had to depend on what primate researchers each deemed “innovative” or “novel,” with little hope of standardizing this. The contrast with the rigorous and prospective collection of relevant data in the study of Perry et al. (2017) could hardly contrast more. More data of this kind will be needed to clarify the significance of juveniles’ innovations.

## Summary and conclusions

As suggested in our title, there is mounting evidence that social learning typically pervades primates’ lifetimes across multiple domains. Of course, this is not to argue that individual-level exploration and learning is unimportant: to the contrary, we have emphasized above a continued alternation and integration of what is acquired through social and asocial learning. We find that the three major phases of social learning that we outlined fit many of the findings available for primates including the great apes and a majority of the monkeys, as well as Strepsirrhine primates where relevant data exist. The third phase following dispersion probably occurs in all species, although in each, just those who migrate. By contrast, the transition between a first phase of learning from primary attachment figures and a subsequent phase of learning from an expanding array of others is likely to be more graded and vary between and within species, in part modulated by variations in competition and tolerance (van Schaik 2003). For example, the unusually committed role of fathers in callitrichidae was mentioned; and just how the ontogeny of social learning is distributed in many other taxa, such as monogamous gibbons, appears to remain largely undocumented. Accordingly, given the patchiness of the data we have been able to draw together in this review, we propose our overarching three-phase scheme should be regarded as a working heuristic hypothesis. We hope that expressed in these tentative terms, our review will help researchers fill the major gaps that still exist in our knowledge of the ontogeny of primate social learning.
